# Research on Assessment Methods for Urban Public Transport Development in China

**DOI:** 10.1155/2014/941347

**Published:** 2014-11-04

**Authors:** Linghong Zou, Hongna Dai, Enjian Yao, Tian Jiang, Hongwei Guo

**Affiliations:** ^1^Graduate School of Architecture, Planning and Preservation, Columbia University, 1172 Amsterdam Avenue, New York, NY 10027, USA; ^2^School of Traffic and Transportation, Beijing Jiaotong University, No. 3 Shang Yuan Cun, Hai Dian District, Beijing 100044, China; ^3^China Urban Sustainable Transport Research Center (CUSTReC), China Academy of Transport Sciences, Ministry of Transport, No. 240 Huixinli, Chaoyang District, Beijing 100029, China; ^4^Department of Transportation Engineering, Beijing Institute of Technology, No. 5 South Zhongguancun Street, Haidian District, Beijing 100081, China

## Abstract

In recent years, with the rapid increase in urban population, the urban travel demands in Chinese cities have been increasing dramatically. As a result, developing comprehensive urban transport systems becomes an inevitable choice to meet the growing urban travel demands. In urban transport systems, public transport plays the leading role to promote sustainable urban development. This paper aims to establish an assessment index system for the development level of urban public transport consisting of a target layer, a criterion layer, and an index layer. Review on existing literature shows that methods used in evaluating urban public transport structure are dominantly qualitative. To overcome this shortcoming, fuzzy mathematics method is used for describing qualitative issues quantitatively, and AHP (analytic hierarchy process) is used to quantify expert's subjective judgment. The assessment model is established based on the fuzzy AHP. The weight of each index is determined through the AHP and the degree of membership of each index through the fuzzy assessment method to obtain the fuzzy synthetic assessment matrix. Finally, a case study is conducted to verify the rationality and practicability of the assessment system and the proposed assessment method.

## 1. Introduction

With the acceleration of the urbanization and the rapid expansion of cities, China has been experiencing a continuous increase in urban population. Consequently, sharp growth in the total travel volume as well as the travel distance of urban residents is witnessed in Chinese cities. As a result, the urban transport structure has undergone significant negative changes: the proportion of motorized travel has risen rapidly while the proportion of nonmotorized travel has gone through a constant decline. These changes raise enormous challenges to Chinese cities with the worsening traffic urban congestion and the rising pressure on environmental pollution and energy consumption. Therefore, the urban transport has become the bottle neck for sustainable urban development. Given the shortage of transport and environmental resources, many issues have to be addressed before an inclusive urban transport system is established in which public transport plays the leading role and the basic travel demands of the wider public are met. These issues include the rapid increase in the number of private automobiles, the deterioration of urban congestion conditions, and the situation where urban public transport plays a passive role in urban development. These make public transport speed up the pace of development to face severe problems.

In an effort of facilitating urban public transport development and improving public transport service capacities, Chinese government has launched the public transport priority development strategy in 2005. The initiative includes the implementation of scientific planning and control, line network optimization, infrastructure construction, and information services improvement as well as other supplementary measures. The goals are to enhance the appeal of public transport systems, reduce the reliance of the public on private automobiles, and improve the operation efficiency of urban transport systems.

In this context, it is of great importance to scientifically assess the development level of urban public transport, identify the gap between urban public transport development and residents' actual travel demands, and reduce the “malposition” effect in urban public transport infrastructure construction and urban development so as to provide a decision-making basis for urban public transport planning, construction, and management in the next step.

During the last two decades, there have been a significant volume of studies assessing public transportation development. Been [1] assessed the quality and quantity of public transport system service. Parkes et al. [2] focused on accessibility. Sheth et al. [3] evaluated bus service level by DEA (data envelopment analysis). Olivková [4] established passenger satisfaction from travel time, regularity and accuracy, time and spatial offer, comfort, costs of freight, and impact on the environment and calculates the average relative importance by passengers who participated in the survey. Vstedal et al. [5] identified 10 key indicators to assess urban public transport accessibility from policy and investment, service operations and standards information, ticketing, vehicles and built environment, and seamless travel. Dodson et al. [6] took multiple indictors into consideration, such as reduction of air pollution, parking congestion mitigation, and reduction of traffic congestion. Mavoaa et al. [7] expanded current public transport accessibility measures by including all components of public transport journey.

In china, a lot of studies have been carried out to assess performance in public transport. Due to the specialty of priority development of urban public transport, synthetic assessment system to evaluate public transport development has yet to be established. Thus, strategic modeling and analysis approaches are needed for evaluating public transport development. In [8, 9], the hierarchical structure for the assessment of urban public transport development level consisting of 23 indexes was constructed in three aspects, namely, infrastructure level, service level, and benefit level. Tian and Wu [10] discussed that the urban public transport development level was assessed from the network structure, infrastructure level, and service level. Wei-Hua et al. [11] considered that the urban public transport development level was assessed synthetically from analysis of convenience, quickness, safety, efficiency, economy, comfort, and environmental influence. In [12], the urban public transport assessment system was established at the network technology level and operation service level; in [13], the implementation effects of public transport priority development measures and plans were assessed from the four aspects of the social economy, transport functions, environment influence, and resource utilization; in [14], the serviceability of urban public transport development was analyzed by introducing the social and economic indexes. The synthetic urban public transport development assessment methods mainly focused on grey cluster method [8, 9, 14], entropy weight matter-element analysis model [4], fuzzy synthetic assessment method [12, 13], multifactor fuzzy assessment [15], and so forth.

In this paper, a multitiered urban public transport development level assessment index system for the conventional bus transport system is established according to the actual conditions of urban public transport development after the implementation of the public transport priority development strategy. A number of complimentary indexes have been proposed additional to the established index system, including public transport prioritized lane ratio, prioritized intersection ratio, harbor-type stop setting rate, and real-time incoming vehicle information forecast coverage, as well as urban and rural passenger transport line transit-oriented operation rate. Finally, the fuzzy AHP combining the AHP and the fuzzy assessment is used to analyze the scientific legitimacy, rationality and serviceability of the assessment index system, and the assessment method based on a case study.

## 2. Establishment of Urban Public Transport Development Assessment Index System

The full-scale implementation of the government initiated strategy of giving priority to urban public transport has given rise to a multitude of prominent characteristics of public transport in large and medium-sized Chinese cities: the acceleration of infrastructure construction, the continuous improvement of public transport service level, the acceleration of IT application, the increasing emphasis on sustainable development, the expanding policy support, and the significant social benefits.

The establishment of a multitiered urban public transport development level assessment index system in this paper is informed by the experiences in implementing the strategy of prioritizing urban public transport. Based on the principle of integrity, scientific legitimacy, comparability, conciseness, and practicality, the assessment index system consisted of a target layer, a criterion layer, and an index layer and evaluates the level of public transit development from six aspects, namely, infrastructure construction level, service level, IT development level, sustainable development level, government support level, and social benefit level (see Table 1). The assessment index system is described as follows.

(*1) Infrastructure Construction Level (U*_1_ *).* Infrastructure construction level reflects macroscopically the rationality of urban public transport planning and construction mainly exploiting the indexes such as tram and bus line network percentage (*U*_11_), bus stop coverage with a radius of 500 m (*U*_12_), dedicated bus lane setting rate (*U*_13_), public transport priority intersection percentage (*U*_14_), bus bay stop setting rate (*U*_15_), and public transport vehicle population per 10,000 persons (*U*_16_). The implementation of prioritizing public transport strategy has accelerated the proliferation of infrastructures which formerly sparsely existed such as bus lanes, bus priority intersections, and harbor-type stops. Therefore, the calculation methods for *U*_13_, *U*_14_, and *U*_15_ are as follows:

(1) $$\begin{array}{c} U_{13}=\frac{l_{bl}}{L},\\ U_{14}=\frac{n_{pj}}{N_{j}},\\ U_{15}=\frac{n_{bbs}}{N_{bs}}, \end{array}$$

where *l*_*bl*_ is length of dedicated bus lanes, *L* is length of bus line networks, *n*_*pj*_ is number of bus priority intersections, *N*_*j*_ is total number of urban trunk intersections, *n*_*bbs*_ is number of bay stops, and *N*_*bs*_ is total number of stops.

(*2) Operation Service Level (U*_2_ *).* As an important manifestation of public transport operation results, the operational level of service reflects the contents such as comfort, punctuality, safety, and public satisfaction of urban public transport system through the indexes such as full load rate of public transport during peak hours (*U*_21_), tram and bus punctuality rate (*U*_22_), average operating speed during peak hours (*U*_23_), tram and bus accident mortality (*U*_24_), public transport passenger satisfaction (*U*_25_), and public transport complaint handling rate (*U*_26_). With the rapid motorization, transport congestion during peak hours worsens at an alerting pace. Meanwhile, the comfort degree of public transport relates directly to the choice of travel mode by the public. Considering these two factors, the calculation method for *U*_21_ is as follows:

(2) $$\begin{array}{c} U_{21}=\frac{\sum_{}^{}n_{p}}{\sum_{}^{}n_{rp}}, \end{array}$$

where *n*_*p*_ is the sum of passengers of all running buses at maximum passenger flow section during peak hours. *n*_*rp*_ is sum of the rated capacity of all running buses at maximum passenger flow section during morning and evening rush hours.

(*3) IT Application Level (U*_3_ *).* IT application, as an important sign of the development level of the modern transport industry, reflects the degree of IT application in urban public transport through the indexes such as installation rate of on-board equipment (*U*_31_), electronic payment card use rate (*U*_32_), and electronic stop board setting rate (*U*_33_). Its calculation method is shown as follows:

(3) $$\begin{array}{c} U_{31}=\frac{n_{vtv}}{N_{v}}\times 100\%,\\ U_{32}=\frac{p_{ec}}{p}\times 100\%,\\ U_{33}=\frac{n_{bf}}{N_{bs}}\times 100\%, \end{array}$$

where *n*_*vtv*_ is the number of vehicles with on-board positioning terminals, *N*_*v*_ is the total number of buses, *p*_*ec*_ is the total number of passengers using electronic payment card, *p* is total number of passengers of public transport, *n*_*bf*_ is total number of stops providing real-time prediction of incoming vehicle information, and *N*_*bs*_ is total number of stops.

(*4) Sustainable Development Level (U*_4_ *).* Sustainable development level sets new requirements for the development of the transport industry. In this paper, sustainable development level reflects the rational use of resources and land of urban public transport systems and their sustainable development level through the indexes such as percentage of green public transport vehicles (*U*_41_) and bus and tram overnight docking rate (*U*_42_). Strengthening the promotion of green public transport vehicles should be an important measure for cities to take the path of sustainable development with low energy consumption, low pollution, and high energy efficiency. The calculation method for *U*_41_ is as follows:

(4) $$\begin{array}{c} U_{41}=\frac{n_{vtv}}{N_{v}}\times 100\%, \end{array}$$

where *n*_*vtv*_ is number of green public transport vehicles and *N*_*v*_ is total number of public transport vehicles.

(*5) Government Support Level (U*_5_ *).* After the implementation of the strategy of giving priority to public transport development, the government has demonstrated expanding support on urban public transport. This paper analyzes the level of government support to urban public transport development from the aspects of subsidy guarantee (*U*_51_) and formulation of relevant policies, laws, and regulations (*U*_52_). The financial aid of the government plays a crucial role in normal operation of public transport, and the calculation method for *U*_51_ in this paper is shown as follows:

(5) $$\begin{array}{c} U_{51}=\frac{M_{ts}}{M_{ps}}\times 100\%, \end{array}$$

where *M*_*ts*_ is actual amount of subsidies for urban public transport; *M*_*ps*_ is total policy subsidies reasonably calculated, including various subsidies calculated by third-sector organizations and undertaken by government, such as new and rare line subsidies, fare subsidies, and mandatory compensation.

(*6) Social Benefits Level (U*_6_ *).* The level of social benefits mainly reflects the positive externalities generated by urban public transport as a public welfare. To reflect such influence, indexes such as number of trips by public transport per capita per day (*U*_61_), mode share of public transport (*U*_62_), and transit-oriented operation rate of urban-rural passenger transport lines (*U*_63_) have been taken into consideration. With the acceleration of urbanization, urban-rural passenger transport lines have gradually adopted the transit-oriented operation mode with low fares, multifrequency, and multistopping sites. The calculation method for *U*_63_ is as follows:

(6) $$\begin{array}{c} U_{63}=\frac{n_{pur}}{N_{ur}}\times 100\%, \end{array}$$

where *n*_*pur*_ is number of urban-rural passenger transport lines adopting the transit-oriented operation mode and *N*_*ur*_ is total number of urban-rural passenger transport lines.

In this paper, the 22 indexes are divided into the 5 grades of “Level 1,” “Level 2,” “Level 3,” “Level 4,” and “Level 5” on the base of “code for transport planning on urban road.” The national standards for actual values of the indexes or the average levels the cities can reach are the upper limit of reference (Table 1).

## 3. Assessment of Urban Public Transport Development Level Based on Fuzzy AHP

Fuzzy mathematics method is applicable for describing qualitative issues by quantitative way, and AHP is able to quantify expert's subjective judgment. Fuzzy AHP is an algorithm proposed to address the problems of the traditional AHP such as difference in judgment consistency and matrix consistency, consistency validation difficulty, and lack of scientificness given the fuzziness in judgment of complicated things [16–19]. It can ensure that the results of assessment of urban public transport development level are objective, impartial, scientific, and reasonable. The fuzzy AHP is to determine the weight of each index through the AHP and establishing the membership function of each index to get the fuzzy synthetic assessment matrix and thus obtain the synthetic assessment values.

### 3.1. Determination of Weight of Each Index through AHP

(*1) Establishment of Judgment Matrix.* To get the local weight in the hierarchical structure of the assessment index system for urban public transport development level, we must gradually establish the judgment matrix. If the upper index *U*_*t*_ contains *n* lower indexes, then *U*_*t*_ can be divided into the following *n* factor sets:

(7) $$\begin{array}{c} U_{t}=\left\{U_{t1},U_{t2},\ldots ,U_{tn}\right\}. \end{array}$$

In this paper, we have adopted 1~9 scales (Table 2), asked the experts to carry out pairwise comparison of the importance of *U*_*ti*_ with respect to *U*_*t*_, and established the judgment matrix *A*_*t*_.

(*2) Consistency Validation of Judgment Matrix and Determination of Weight Set.* The root method is used to calculate the maximal eigenvector of the judgment matrix. If the judgment matrix *A*_*t*_ satisfies the consistency requirements, its eigenvector [*w*_*t*1_, *w*_*t*2_,…, *w*_*tn*_] corresponding to the maximum eigenvalue should be served as the weight coefficient; if the judgment matrix *A*_*t*_ dissatisfies with the consistency requirements, it should be properly adjusted. The adjustment method can be referred to in [20].

For this paper, experts have been invited to carry out pairwise comparison according to the standards in Table 2 and the AHP judgment matrix has been obtained. The weights of each index and the weighted average have been obtained, and the weight vectors obtained are as shown in Table 3.

### 3.2. Establishment of Fuzzy Assessment Matrix

(*1) Establishment of Judgment Set.* The grading is carried out according to the indexes in the assessment system that can be divided into the 5 grading criteria of “Level 1,” “Level 2,” “Level 3,” “Level 4,” and “Level 5.” Let the judgment set be *V* = {*V*_1_, *V*_2_, *V*_3_, *V*_4_, *V*_5_}, in which *V*_*i*_  (*i* = 1,2, 3,4, 5) means the *i*th grade.

(*2) Determination of Membership Degree.* To better reflect the characteristics of each assessment index of “Transit Metropolis,” we eliminate the dimensional effect of each assessment index, and it is necessary to carry out the abstract and fuzzy processing of each index data.

For the quantitative indexes in this system, the following membership function is adopted in this paper for smaller better indexes, such as full load rate of public transport during peak hours and tram and bus accident mortality:

(8) $$\begin{array}{c} r\left(i\right)=\left\{\begin{array}{cc} 1, & x\left(i\right)<a,\\ \frac{b-x\left(i\right)}{b-a}, & a\leq x\left(i\right)<b,\\ 0, & x\left(i\right)\geq b, \end{array}\right. \end{array}$$

where *r*(*i*) is membership function of index *i*; *x*(*i*) is the actual values of index *i*;  *a*,  *b* are the upper and lower boundaries of membership function.

For bigger better indexes, such as bus stop coverage with a radius of 500 m and dedicated bus lane setting rate, the following membership function can be adopted:

(9) $$\begin{array}{c} r\left(i\right)=\left\{\begin{array}{cc} 0, & x\left(i\right)<a,\\ \frac{x\left(i\right)-a}{b-a}, & a\leq x\left(i\right)<b,\\ 1, & x\left(i\right)\geq b. \end{array}\right. \end{array}$$

For the qualitative indexes, such as complete degree of relevant policies, laws, and regulations, their membership degree can be determined according to the fuzzy statistical method [16].

(*3) Establishment of Fuzzy Assessment Matrix of Indexes.* The single factor assessment matrix of the *i*th subset of the urban public transport development level assessment index system is established as follows: *r*_*ti*_ = {*r*_*ti*1_, *r*_*ti*2_,…, *r*_*ti*5_},  *i* = 1,2,…, *n*, in which *r*_*tij*_  (*i* = 1,2,…, *n*; *j* = 1,2,…, 5) means the membership degree of the *ij* lower index of the index *U*_*t*_ with respect to the grade *V*, and the matrix *r*_*tij*_ is normalized to obtain the fuzzy assessment matrix *R*_*t*_ of the index *U*_*t*_:

(10) $$\begin{array}{c} R_{tn\times 5}=\left[\begin{array}{ccc} r_{ti1}^{\prime } & \cdots & r_{ti5}^{\prime }\\ \vdots & \ddots & \vdots \\ r_{tn1}^{\prime } & \cdots & r_{tn5}^{\prime } \end{array}\right]. \end{array}$$

### 3.3. Analysis of Synthetic Assessment Results

The results of synthetic assessment are obtained through compound calculation of the single factor weight matrix *w*_*t*_ and the fuzzy matrix *R*_*t*_, that is, the synthetic assessment set *U*_*t*_, which is denoted as follows:

(11) $$\begin{array}{c} U_{t}=w_{t}\times R_{t}=\left(U_{t1},U_{t2},\ldots ,U_{t5}\right). \end{array}$$

The synthetic assessment matrix *U* of urban public transport development level can eventually be obtained through calculating gradually upwards layer by layer from the index layer. According to the principle of maximum degree of membership, the assessment grade corresponding to the maximum element in the matrix *U* is the synthetic assessment grade of urban public transport development level.

The score of the determined assessment grade is written in the following vector calculation form:

(12) $$\begin{array}{c} Q=\left[100,80,70,60,40\right]. \end{array}$$

The total score *S* of the assessment index system can be obtained by multiplying *U* and *Q*:

(13) $$\begin{array}{c} S=U*Q^{T}. \end{array}$$

## 4. Case Study

Kunming is a major city in southwest China. Along with the rapid advancement of socioeconomic development as well as fast urbanization, the city is featured by the adjustment of urban spatial structure, the continuous improvement of transport infrastructure, the sustainably growth in vehicle numbers, and the drastic increase in residents' trip demands. Those factors have led to the increasingly serious congestion in the city center. As Kunming is one of the first batch cities in the “Transit Metropolis” Program announced by the Ministry of Transport of China, its urban public transport development has attracted increasing professional as well as academic attention. The present public transport development of Kunming is analyzed by using the fuzzy AHP as the following.

(*1) Establishment of Membership Matrix.* The related assessment data is acquired according to the data on Kunming's urban public transport development in 2011 (Table 4).

The membership degree matrix is calculated and normalized according to the index grading criteria in Table 1, and the fuzzy assessment subsets of the 22 indexes are acquired, which form 6 fuzzy assessment matrixes *R*_1_, *R*_2_, *R*_3_, *R*_4_, *R*_5_, and *R*_6_ corresponding to the indexes at the criteria layer:

(14) $$\begin{array}{c} R_{1}=\left[\begin{array}{ccccc} 0 & 0 & 0.094 & 0.5 & 0.406\\ 0 & 0 & 0.22 & 0.5 & 0.28\\ 0 & 0.33 & 0.5 & 0.17 & 0\\ 0 & 0 & 0.167 & 0.5 & 0.333\\ 0 & 0.075 & 0.5 & 0.425 & 0\\ 0 & 0.284 & 0.5 & 0.216 & 0 \end{array}\right],\\ R_{2}=\left[\begin{array}{ccccc} 0 & 0 & 0.05 & 0.5 & 0.45\\ 0 & 0 & 0 & 0.313 & 0.687\\ 0 & 0.425 & 0.5 & 0.075 & 0\\ 0 & 0.3 & 0.5 & 0.2 & 0\\ 0.5 & 0.5 & 0 & 0 & 0\\ 0.526 & 0.474 & 0 & 0 & 0 \end{array}\right],\\ R_{3}=\left[\begin{array}{ccccc} 0 & 0 & 0 & 0.526 & 0.474\\ 0 & 0 & 0.5 & 0.5 & 0\\ 0 & 0 & 0 & 0 & 1 \end{array}\right],\\ R_{4}=\left[\begin{array}{ccccc} 0 & 0 & 0 & 0.195 & 0.805\\ 0.844 & 0.156 & 0 & 0 & 0 \end{array}\right],\\ R_{5}=\left[\begin{array}{ccccc} 0.2 & 0.5 & 0.3 & 0 & 0\\ 0.1 & 0.2 & 0.6 & 0.1 & 0 \end{array}\right],\\ R_{6}=\left[\begin{array}{ccccc} 0 & 0 & 0 & 0.448 & 0.552\\ 0 & 0.425 & 0.5 & 0.075 & 0\\ 0.475 & 0.5 & 0.025 & 0 & 0 \end{array}\right]. \end{array}$$

(*2) Calculation and Analysis of Assessment Results.* According the principle of maximum membership degree, the maximum value in fuzzy set is the evaluation result. The fuzzy synthetic assessment results show that the synthetic assessment result of Kunming's urban public transport development is “Level 3,” and the overall score is 66.53, so its public transport system can be considered to be reasonable and meet the actual conditions (Table 5).

The fuzzy assessment matrix and the synthetic assessment result show that there are still some problems existing in the development of urban public transport in Kunming.

1. The infrastructures need to be improved markedly. The pace of urban public transport infrastructure construction is not in line with that of urban construction, and the percentage of bus line network is still at a very low level of only 36.87%; the dedicated bus lane setting rate is 13.3%; the bus priority intersection rate is 10%; the bus bay stop setting rate is 13%; and the public transport vehicle population per 10,000 persons is 13.7 standard units, mostly in the moderate or poor grades. In the future, it is necessary to increase the input in public transport-dedicated road facility construction and transport capacity improvement to enhance the appeal of public transport.
2. The level of IT application remains to be improved. The level of digitization and intellectualization of urban public transport in Kunming needs to be improved, which is the direction of urban public transport development in the next step. In addition, it is necessary to strengthen the input in intelligent on-board electronic devices, to constantly improve the use rate of card for bus riding and to make a breakthrough in the “zero stage” situation in electronic bus stop board services to make it convenient for the public to travel and attract more passengers to travel by public transport systems.
3. The level of sustainable development needs to be increased. Based on the requirements of resource conservation and environmental protection, it is necessary to promote the development of new energy vehicles for public transport with emphasis on energy conservation and emission reduction. Additionally, the phase-out of old vehicles should speed up to improve the overall transport capacity. The priorities of land use for bus stops should be ensured to address the problem of public transport vehicle parking.
4. The guarantee and support from the government need to be materialized. Public transport will be difficult to operate normally without the policy support and investment of the government. In the future, public transport vehicles, road infrastructures, and IT application still need considerable finance-policy support; it has become a key task of how to ensure various bus priority policies implementation of the government in the next step.

## 5. Conclusions

This paper establishes a multitiered urban public transport development assessment system, in which the priorities are given to public transport according to the characteristics of public transport development in medium and large cities in China. Some of important assessment indexes were taken into consideration including the infrastructure construction, the service level of public transport, the acceleration of IT application, and the increasing emphasis on sustainable development, as well as the expanding policy support and significant social benefits. The assessment model is hence established based on the fuzzy AHP. The weight of each index is determined through the AHP and the degree of membership of each index is calculated through the fuzzy assessment method to obtain the fuzzy synthetic assessment matrix. With such methodology, the overall assessment score of urban public transport development level can be analyzed quantitatively. In addition, Kunming, China, is studied as an example to prove the rationality and practicability of the assessment system and the assessment method.

The results from the case study show that a quantitative assessment can be obtained to directly reveal the actual development of public transport in Kunming city. However, due to the limit of resources and scopes, the case study uses the data from only one city, which makes it hardly a comprehensive contrastive analysis. In future work, the contrastive analysis will be performed by using the data derived from multiple cities of comparable scale and economic level, which would be an essential work to test and verify the proposed model.

## Figures and Tables

**Table 1:** Urban public transport development level assessment system and grading criteria.

Target layer	Criterion layer	Index layer	Unit	Grading criteria				
				Level 1	Level 2	Level 3	Level 4	Level 5
Urban public transport development level assessment system *U*	Infrastructure construction level *U*_1_	Tram and bus line network percentage *U*_11_	%	≥65	[55, 65)	[45, 55)	[35, 45)	<35
		Bus stop coverage with the radius of 500 m *U*_12_	%	≥95	[90, 95)	[85, 90)	[80, 85)	<80
		Rate of dedicated bus lane *U*_13_	%	≥20	[15, 30)	[10, 15)	[5, 10)	<5
		The percentage of intersections with public transport priority *U*_14_	%	≥50	[30, 50)	[10, 30)	[5, 10)	<5
		Rate of setting bus and tram bay stop *U*_15_	%	≥50	[30, 50)	[10, 30)	[5, 10)	<5
		Public transport vehicles per 10,000 persons *U*_16_	Units/10000 persons	≥18	[15, 18)	[12, 15)	[10, 12)	<10
	Operation service level *U*_2_	Full load rate of public transport during peak hours *U*_21_	%	[60, 90)	[90, 100)	[100, 110)	[110, 120)	≥120
		Tram and bus punctuality rate *U*_22_	%	≥75	[70, 75)	[65, 70)	[60, 65)	<60
		Average operating speed during peak hours *U*_23_	km/h	≥19	[17, 19)	[15, 17)	[13, 15)	<13
		Tram and bus accident mortality *U*_24_	Pass/million km	<0.03	[0.03, 0.035)	[0.035, 0.04)	[0.04, 0.045)	[0.045, 0.05)
		Public transport passenger satisfaction *U*_25_	%	≥80	[75, 80)	[70, 75)	[65, 70)	<65
		Public transport complaint handling rate *U*_26_	%	≥80	[70, 80)	[60, 70)	[50, 60)	<50
	IT application level *U*_3_	Installation rate of electronic on-board positioning terminal *U*_31_	%	≥95	[85, 95)	[75, 85)	[65, 75)	<65
		Electronic payment card use rate *U*_32_	%	≥70	[60, 70)	[50, 60)	[40, 50)	<40
		Coverage of incoming vehicle *i* real-time forecast *U*_33_	%	≥75	[50, 75)	[30, 50)	[20, 30)	<20
	Sustainable development level *U*_4_	Percentage of green public transport vehicles *U*_41_	%	≥60	[50, 60)	[40, 50)	[30, 40)	<30
		Docking rate of bus and tram during night *U*_42_	%	≥90	[80, 90)	[70, 80)	[60, 70)	<60
	Government support level *U*_5_	Availability of subsidy for public transport operation *U*_51_	%	≥75	[50, 75)	[40, 50)	[20, 40)	<20
		Complete degree of related policies, laws, and regulations *U*_52_		Level 1	Level 2	Level 3	Level 4	Level 5
	Social benefit level *U*_6_	Number of trips by public transport per capita per day *U*_61_	Times	≥1.04	[0.9, 1.04)	[0.77, 0.9)	[0.63, 0.77)	<0.63
		Model share of public transport *U*_62_	%	≥60	[50, 60)	[40, 50)	[30, 40)	<30
		Transit-oriented operation rate of urban-rural passenger transport lines *U*_63_	%	≥85	[75, 85)	[65, 75)	[55, 65)	<55

**Table 2:** Explanations of 1~9 scales.

Value	Explanation
1	Two indexes are equally important.
3	By comparison, *x*_*i*_ is slightly more important than *x*_*j*_.
5	In comparison, *x*_*i*_ is clearly more important than *x*_*j*_.
7	In comparison, *x*_*i*_ is much more important than *x*_*j*_.
9	In comparison, *x*_*i*_ is extremely more important than *x*_*j*_.
2, 4, 6, 8	Intermediate state corresponding to the above adjacent judgment.

**Table 3:** Weight vectors of assessment indexes.

Weight vector	Results
*w*	[0.294, 0.245, 0.0951, 0.0814, 0.1591, 0.1254]
*w*_1_	[0.2411, 0.3547, 0.1078, 0.0961, 0.067, 0.1333]
*w*_2_	[0.1011, 0.1719, 0.2105, 0.1887, 0.225, 0.1028]
*w*_3_	[0.4773, 0.2958, 0.2269]
*w*_4_	[0.6389, 0.3611]
*w*_5_	[0.6574, 0.3426]
*w*_6_	[0.4352, 0.3686, 0.1962]

**Table 4:** Basic data on urban public transport of Kunming.

Index	Value
Percentage of tram and bus line network (%)	36.87
Bus stop coverage with a radius of 500 m (%)	82.20
Rate of setting dedicated bus lane setting (%)	13.30
The percentage of intersections with public transport priority (%)	10
Rate of setting bus and tram bay stop (%)	13
Public transport vehicle per 10,000 persons (units/10,000 persons)	13.7
Full load rate of public transport during peak hours (%)	92
Tram and bus punctuality rate (%)	45.46
Average operating speed during peak hours (km/h)	15.7
Tram and bus accident mortality (pass/million km)	0.037
Public transport passenger satisfaction (%)	80
Public transport complaint handling rate (%)	82
Installation rate of electronic on-board positioning terminal equipment (%)	58.60
Electronic payment card use rate (%)	50
Incoming vehicle information real-time forecast coverage (%)	0
Percentage of green public transport vehicles (%)	7.30
Bus and tram overnight docking rate (%)	96.30
Availability of public transport operation subsidy (%)	60
Number of trips by public transport per capita per day (times)	0.51
Model sharing of public transport (%)	48.50
Transit-oriented operation rate of urban-rural passenger transport lines (%)	84.50

**Table 5:** Assessment result of urban public transport development level of Kunming.

Assessment target	Fuzzy assessment matrix	Assessment results	Value
Infrastructure construction level	[0, 0.0785, 0.2708, 0.4215, 0.2292]	Level 4	59.69
Operation service level	[0.1666, 0.3073, 0.2047, 0.1579, 0.1636]	Level 2	71.59
IT application level	[0, 0, 0.1479, 0.3990, 0.4531]	Level 5	52.97
Sustainable development level	[0.3048, 0.0563, 0, 0.1246, 0.5143]	Level 5	63.03
Government support level	[0.1657, 0.3972, 0.4028, 0.0343, 0]	Level 3	78.60
Social benefit level	[0.0932, 0.2548, 0.1892, 0.2226, 0.2402]	Level 2	65.91
Overall assessment index	[0.1037, 0.2076, 0.2407, 0.2346, 0.2134]	Level 3	66.53

## References

[1] Been H. P. (1995). *Bus Route Evalution Standards*.

[2] Parkes T., Corcoran B., Gould J. (2003). *Evaluation of the Act Action Plan for Accessible Public Transport*.

[3] Sheth C., Triantis K., Teodorović D. (2007). Performance evaluation of bus routes: a provider and passenger perspective. *Transportation Research Part E: Logistics and Transportation Review*.

[4] Olivková I. (2010). *Evaluation of Quality Indicators Public Transport. Number IV*.

[5] Vstedal L., Azalde G., Derud T. Accessibility indicators for urban public transport.

[6] Dodson J., Mees P., Stone J., Burke M. (2011). *The Principles of Public Transport Network Planning: A Review of the Emerging Literature with Select Examples: Urban Research Program*.

[7] Mavoaa S., Wittena K., McCreanorb T., O’Sullivanc D. (2012). GIS based destination accessibility via public transit and walking in Auckland, New Zealand. *New Zealand. Journal of transport geography*.

[8] Yuhua L., Kai S. (2004). On the comprehensive evaluation of urban public transport based on gray clustering method. *Journal of Harbin University*.

[9] Yuhua L., Yunquan H. (2006). Gray clustering method applied to evaluation of Urban public transport development level. *Mathematics in Practice and Theory*.

[10] Tian M., Wu Z. (2010). Fuzzy comprehensive evaluation for Urban public transport development. *Urban Public Transportation*.

[11] Wei-Hua Z., Hua-Pu L., Qiang L. (2004). Study on evaluation index system of bus priority measures. *Communications Standardization Issue*.

[12] Liu Z., Yu Y., Cheng S. (2010). A comprehensive evaluation index system for public transportation based on entropy weight matter-element model. *Urban Transport of China*.

[13] Cai W.-X., Guo X.-T. The research & evaluation of urban and rural public transport networks based on AHP and the fuzzy comprehensive evaluation.

[14] Chen H., Wu Y., Guo Y. Adaptability evaluation of urban public transport based on weighted grey incidence degree.

[15] Xu Y., Qiao J., Wang L. Application of multi-factor hierarchical fuzzy evaluation in public transport.

[16] Wang Q., Wen F., Liu M., Yi S. (2009). Combined use of fuzzy set theory and analytic hierarchy process for comprehensive assessment of electricity markets. *Automation of Electric Power Systems*.

[17] Chan Z., Xin L., Tongtao L. (2009). Research on comprehensive evaluation of network performance based on FAHP. *Application Research of Computer*.

[18] Chen L. (2011). Application of fuzzy analytical hierarchy process in airline safety evaluation. *Aeronautical Computing Technique*.

[19] Saaty T. L., Tran L. T. (2007). On the invalidity of fuzzifying numerical judgments in the analytic hierarchy process. *Mathematical and Computer Modelling*.

[20] Satty T. L. (1980). *The Analytic Hierarchy Process*.

